# Regional differences and temporal trend analysis of Hepatitis B in Brazil

**DOI:** 10.1186/s12889-022-14296-1

**Published:** 2022-10-17

**Authors:** Giuliano Grandi, Luis Fernandez Lopez, Marcelo Nascimento Burattini

**Affiliations:** 1 Infectious Diseases Division, Escola Paulista de Medicina, Hospital São Paulo, Universidade Federal de São Paulo, São Paulo, SP Brazil; 2 Institute of Mathematics and Statistics, The University of São Paulo, São Paulo, SP Brazil; 3 Discipline of Medical Informatics and LIM-01 HCFMUSP, School of Medicine, The University of São Paulo, São Paulo, SP Brazil; 4 Center for Internet Augmented Research and Assessment - CIARA, Florida International University, Florida, USA; 5 Present Address: Rua Botucatu, 740–5th floor. Room 507, CEP 04023-062 São Paulo, SP Brazil

**Keywords:** Hepatitis B virus, Hepatitis B, Epidemiologic methods, Epidemiological monitoring

## Abstract

**Background:**

Burden disease related to chronic HBV infection is increasing worldwide. Monitoring Hepatitis B occurrence is difficult due to intrinsic characteristics of the infection, nonetheless analyzing this information improves strategic planning towards reducing the burden related to chronic infection. In this line of thought, this study aims to analyze national and regional epidemiology of Hepatitis B and it’s temporal trends based on Brazilian reported cases.

**Methods:**

Data obtained from the Brazilian National Notifiable Disease Reporting System (SINAN) from 2007 to 2018 were classified by infection status with an original classification algorithm, had their temporal trends analyzed by Joinpoint regression model and were correlated with gender, age and region.

**Results:**

Of the 487,180 hepatitis B cases notified to SINAN, 97.65% had it infection status correctly classified by the new algorithm. Hepatitis B detection rate, gender and age-distribution were different among Brazilian regions. Overall, detection rates remained stable from 2007 to 2018, achieving their maximal value (56.1 cases per 100,000 inhabitants) in North region. However, there were different temporal trends related to different hepatitis B status and age. Women mean age at notification were always inferior to those of men and the difference was higher in Central-West, North and Northeast regions.

**Conclusion:**

Hepatitis B affects heterogeneously different populations throughout Brazilian territory. The differences shown in its temporal trends, regional, gender and age-related distribution helps the planning and evaluation of control measures in Brazil.

**Supplementary Information:**

The online version contains supplementary material available at 10.1186/s12889-022-14296-1.

## Introduction

Viral hepatitis has significant worldwide burden, with increasing associated mortality. The Global Health Sector Strategy, states that understanding viral hepatitis epidemiology is key to the goal of eliminating it by 2030 [1].

The Global Burden Disease Study analysis [2], showed that the number of hepatitis related deaths increased 63% (870,000 to 1,450,000) from 1997 to 2013. The hepatitis associated deaths in 2015 had chronic liver disease and primary liver cancer as their leading causes [3, 4]. Therefore, chronic Hepatitis B and C plays a major role in viral hepatitis burden.

Notwithstanding, good estimates of Hepatitis B Virus (HBV) infection rate are difficult due to the lack of good quality data and to the high frequency of asymptomatic or long-term chronically infected cases [5, 6].

In order to analyze population features of infectious diseases, one must define the scale of the study. Local or specific population driven studies are more suitable to characterize risk factors related to the infection [7–10], while national or regional population studies can identify major patterns related to socio-demographic characteristics of a region in order to compare it to other world regions. This manuscript follows this last stream of thought.

**Fig. 1:**
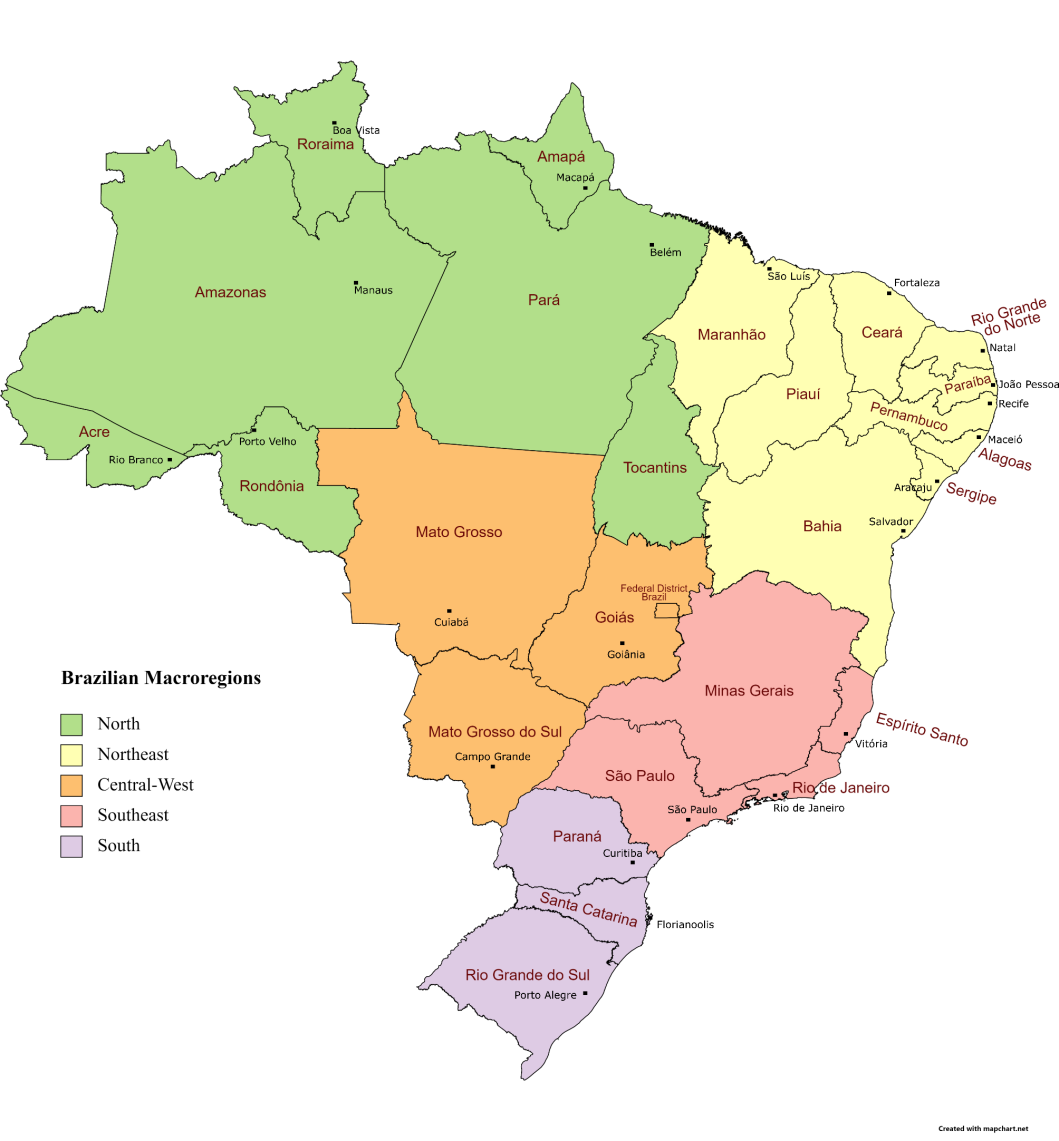
Geopolitical map of Brazil with Macroregions, state names (red) and capital cities names (black)

Brazil is the fifth largest country in the world in terms of territory and population size (209,096,705) [11]. It’s divided in 5 geographical regions (Fig. 1) – Southeast (4 states), with population of 87,521,315; Northeast (9 states), population of 57,576,309; South (3 states), population of 29,754,036; North (7 states), with population of 18,158,149; Central-West (3 states and the Federal District), population of 16,086,896 - with heterogeneous socio-demographic characteristics and Hepatitis B cases distribution [12, 13].

The 1988 Brazilian Constitution state that health assistance to the diseased is responsibility of Brazilian Ministry of Health (MoH), which incorporates an unified and nationwide health care system (Sistema Único de Saúde - SUS). The Health Surveillance Secretary is in charge of promoting surveillance and orienting health assistance and control strategic planning to decrease transmission of communicable diseases [14, 15]. The Department of Chronic Illness and Sexually Transmitted Infections (Departamento de Doenças de Condições Crônicas e Infecções Sexualmente Transmissíveis – DCCI) is responsible for developing strategies to promote health assistance and decrease transmission of HIV, viral hepatitis and sexually transmitted diseases [15].

The National Reportable Disease Information System (Sistema de Informação de Agravos de Notificação - SINAN), created in 1999, after the National System for Epidemiologic Surveillance (Sistema Nacional de Vigilância Epidemiológica - created in 1976), receives data of compulsorily notifiable diseases cases, which includes viral hepatitis [15, 16]. Clinical, demographic, epidemiologic and laboratory data provide the basis for the investigation of suspected cases on the SINAN database repositories. SINAN allows the identification of a health condition or illness occurrence at individual level, therefore allowing the study and interpretation of related epidemiologic conditions in any given Brazilian geographic region. This database comprises two parts [15, 16].

- Individual Notification, including socio-demographic data pertaining to individuals and applying to all compulsorily diseases notifiable via SINAN;
- An epidemiological form, specific to each compulsorily notifiable disease or condition, including clinic, epidemiologic and laboratory data specific to it.

The hepatitis surveillance system, launched with SINAN in 1999, provides nationwide clinical-demographic-epidemiologic data related to viral hepatitis including Hepatitis B. In this manuscript we analyze B hepatitis data from a SINAN extracted database, providing a detailed description of Hepatitis B occurrence, as related to its temporal trends, age, gender and regional characteristics in Brazil, discussing socio-demographic-epidemiologic determinants of its burden throughout the country.

## Methods

Hepatitis B data, from 2007 to 2018, extracted from SINAN database (SINAN Net – Version 5.0) anonymized, cleaned, reviewed and consisted constituted the study database. This study used the following SINAN Viral Hepatitis variables:

- A study defined indexing number – serving as an index variable for the database;
- dates of birth, first symptoms and notification;
- state of notification;
- gender;
- serological markers of HBV infection – Anti-HBs, HBsAg, Anti-HBe, HBeAg, Anti-HBc total and Anti-HBc IgM – referred to as Reagent, Not-reagent, Undetermined and Not realized.

Two other variables aiming to classify Hepatitis B infection status were included in the database. First, ***HBV Class 1***, following the current Brazilian and European recommendations for HBV case definition [17]. Second, ***HBV Class 2***, originally proposed here, modifying ***HBV Class 1*** definition to make it more congruent with the actual notification practice in Brazil. The proposed ***HBV Class 2*** definition is:

- Infected: any serological marker for HBV infection;
- Acute: HBsAg positive **and** Anti-HBc IgM positive;
- Chronic: HBsAg positive **and** (Anti-HBs negative **or** undetermined **or** not informed);
- Resolved: (Anti HBc total positive **or** Anti HBs positive) **and** (HBsAg negative **or** undetermined **or** not informed).

The definitions and agreement between both variables are shown in Tables 1 and 2.

**Table 1:** Description of two different HBV case definitions based on serological markers. HBV Class 1 refers to international HBV status classification. HBV Class 2 refers to a modified classification proposed by the authors in order to maximize available data for analysis

	HBV Class 1	HBV Class 2
HBV Infection	Any serological marker for HBV Infection	Any serological marker for HBV Infection
Acute	HBsAg positive **and** Anti-HBc IgM positive **and** Anti-HBc total positive	HBsAg positive **and** Anti-HBc IgM positive
Chronic	HBsAg positive **and** Anti-HBc IgM negative **and** Anti-HBc total positive	HBsAg positive **and** (Anti-HBs negative **or** undetermined **or** not informed)
Resolved	Anti-HBs positive **and** Anti-HBc total positive	(Anti-HBc total positive **or** Anti-HBs positive) **and** (HBsAg negative **or** undetermined **or** not informed)

### Concordance analysis

Kappa analysis compared the agreement between both case definitions, ***HBV Class 1*** and ***HBV Class 2***.

### Statistical analysis

Population age-stratified data [11] allowed calculation of the yearly national and regional notification rates of HBV *Infected*, *Acute*, *Chronic* and *Resolved* cases per 100,000 inhabitants.

Brazilian and regional annual notification rates calculated for each 10 years age interval (between 1 and 89 years) by gender and infection status allowed the analyses of absolute and age-related temporal trends.

Data fitted to a Joinpoint Regression Model selected by Bayesian Information Criteria (BIC) [18] allowed the calculation of the Annual Percent Change (APC), when one or two joinpoints were identified, and the calculation of the Average Annual Percent Change (AAPC) for the whole period [19] as surrogates for the dynamics of HBV incidence in Brazil. Results related to APC or AAPC, expressed as percentage with 95% CI and written as (APC or AAPC: -22.1%; -35% to -6.7%), describe the findings. To describe trends, the terms ‘increase’ and ‘decrease’ were used when AAPC or APC achieved statistical significance (*p* < 0.05), otherwise the term ‘stable’ was used.

The Welch Two Sample T-test for means with unknown variances compared gender differences of the mean age at notification among different Brazilian regions. In addition, One-way ANOVA with Bonferroni tests compared regional differences on the mean age at notification by gender. Together, both analysis allowed a better description of the different regional patterns of HBV population dynamics in Brazil. Statistical significance level was set at 5% (a = 0.05). The TBCO Statistica 13.5.0.17 and the Joinpoint Trend Analysis 4.9.1.0 software were used for analysis.

## Results

### Classification analysis

In the 2007–2018 period, 487,179 identified cases of HBV infection were included in the analysis. ***HBV Class 1*** classified HBV infection status of only 237,034 cases (48.65%), while ***HBV Class 2*** allowed the identification of HBV infection status in 475,759 cases (97.65%). See Table 2 for details.

#### Concordance analysis

Concordance analysis demonstrated only a poor agreement between both case definitions criteria, with a *Kappa* value of 0.312 (95% CI: 0.311 to 0.314) when considering all cases in the database, including those non-classified by either classification variable. However, this poor agreement mainly reflects the lack of classified cases by ***HBV Class 1*** (242,845).

When considering only cases simultaneously classified by both variables (232,915), the agreement was perfect (*Kappa = 1.0*), meaning that ***HBV Class 2*** correctly classified all cases classified by ***HBV Class 1***.

In addition, the 4.119 cases classified as *Resolved* by ***HBV Class 1*** and Not Classified by ***HBV Class 2*** probably reflect miss interpretation. The simultaneous result of ***HBsAg and Anti-HBs positivity*** seen in all of them should not allow their classification as *Resolved* (***HBV Class 1*** takes into account only *Anti-HBs positivity*), unless interpreted in association with other serological, pathological, molecular or clinic-epidemiological markers, as the presence of HBsAg positivity should preclude the classification of *Resolved*. Table 2 summarizes the results used on this agreement analysis.

**Table 2:** Comparison of **HBV Class 1** and **HBV Class 2** performance using data available at SINAN database. **(a)** Shows the number of cases classified by each classification system, and the differences between them. **(b)** Shows the agreement between cases classified by both classification systems

**a)**		**HBV Class 1**	**HBV Class 2**	**Difference (Class 2 − 1)**		
	Acute	9.93	13.669	3.739		
	Chronic	57.616	161.283	103.667		
	Resolved	169.488	300.807	131.319		
	Not classified	250.146	11.42	-238.726		
**b)**	HBV Class 2					
		**Acute**	**Chronic**	**Resolved**	**Not classified**	**All groups**
	Acute	9.93	0	0	0	9.93
	Chronic	0	57.616	0	0	57.616
HBV Class 1	Resolved	0	0	165.369	4.119	169.488
	Not classified	3.739	103.667	135.438	7.301	250.146
	All groups	13.669	161.283	300.807	11.42	487.179

#### Trend analysis

Trend analysis showed that the incidence of Brazilian HBV *Infected*, *Chronic* and *Resolved* cases remained stable, but decreased for *Acute* cases, from 2007 to 2018. Trend analysis grouped by age intervals showed that the incidence of HBV *Infected* cases in Brazil decreased from 2007 to 2018 for the ages 1–9, 10–19 and 20–29, remaining stable for the others.

For *Acute* cases, the incidence decreased from 2007 to 2018 for all 10-years age intervals between 1 and 9 years and 30–39 years. For ages above 40 and bellow 60 years old, the incidence remained.

The incidence of *Chronic* cases decreased from 2007 to 2018 for the ages bellow 30 years, and increased for those older than 40. Finally, the incidence of *Resolved* cases decreased for ages bellow 50.

**Fig. 2:**
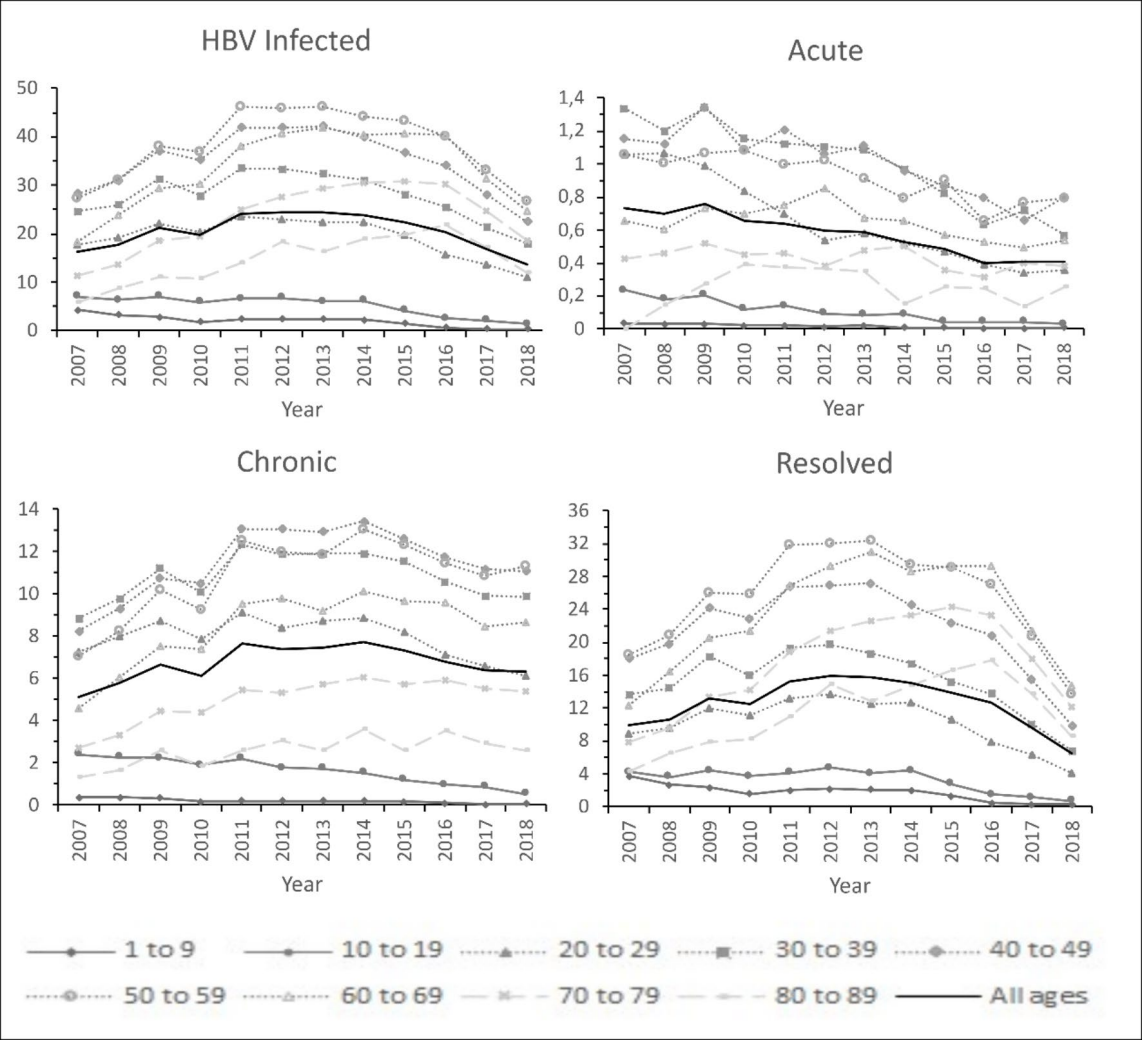
Reported incidence trends of Hepatitis B cases per 100,000 inhabitants in Brazil, by case definition and age group from 2007 to 2018

Figure 2 illustrates the findings related to temporal analysis while AAPC details can be found in table S1 of the Supplementary Material.

#### Gender and Regional differences

Hepatitis B distribution is heterogeneous in Brazil. From 2007 to 2018 the Southeast region notified 218,320 (45.88%), South 96,215 (20.22%), North 73,474 (15.44%), Central-West 49,323 (10.36%) and Northeast 38,427 (8.1%).

**Fig. 3:**
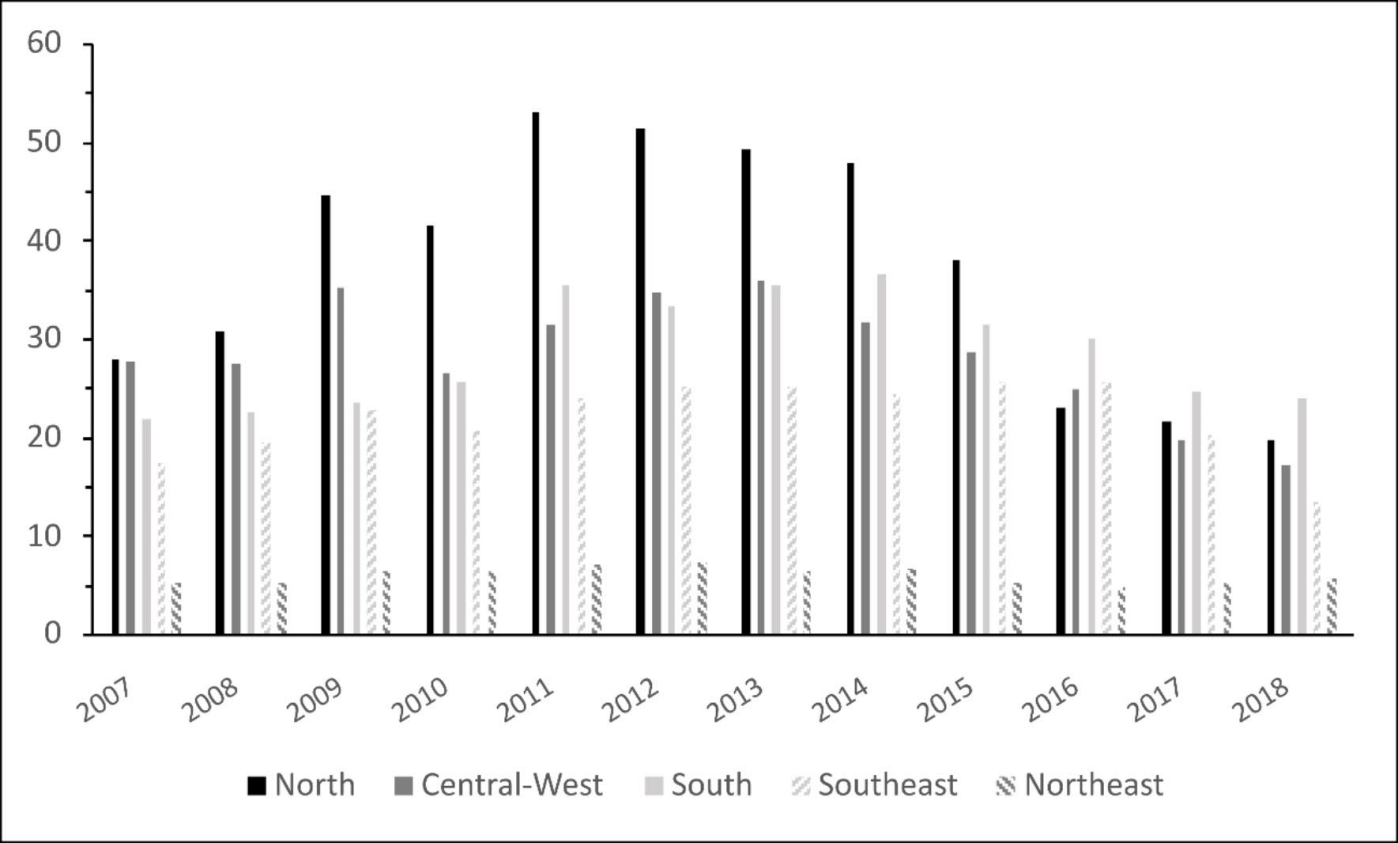
Detection rate of Hepatitis B cases per 100,000 inhabitants, by year and Brazilian region

In contrast, the North region reported the largest *Infected* from 2007 to 2015, peaking at 53.01 cases per 100,000 inhabitants in 2011. However, from 2011 onwards its incidence consistently decreased, being surpassed by region South from 2016 on, as shown in Fig. 3.

Another aspect worth mentioning is the higher proportion of *Chronic* HBV cases notified in South (52.74%; 95% CI: 52.42–53.05) and Northeast (41.3%; 95% CI: 40.81–41.79) regions, as compared to its proportion in Brazil (33.9%; 95% CI: 33.7–34.03). Table S2 in Supplementary Material exhibits details of this analysis.

**Fig. 4:**
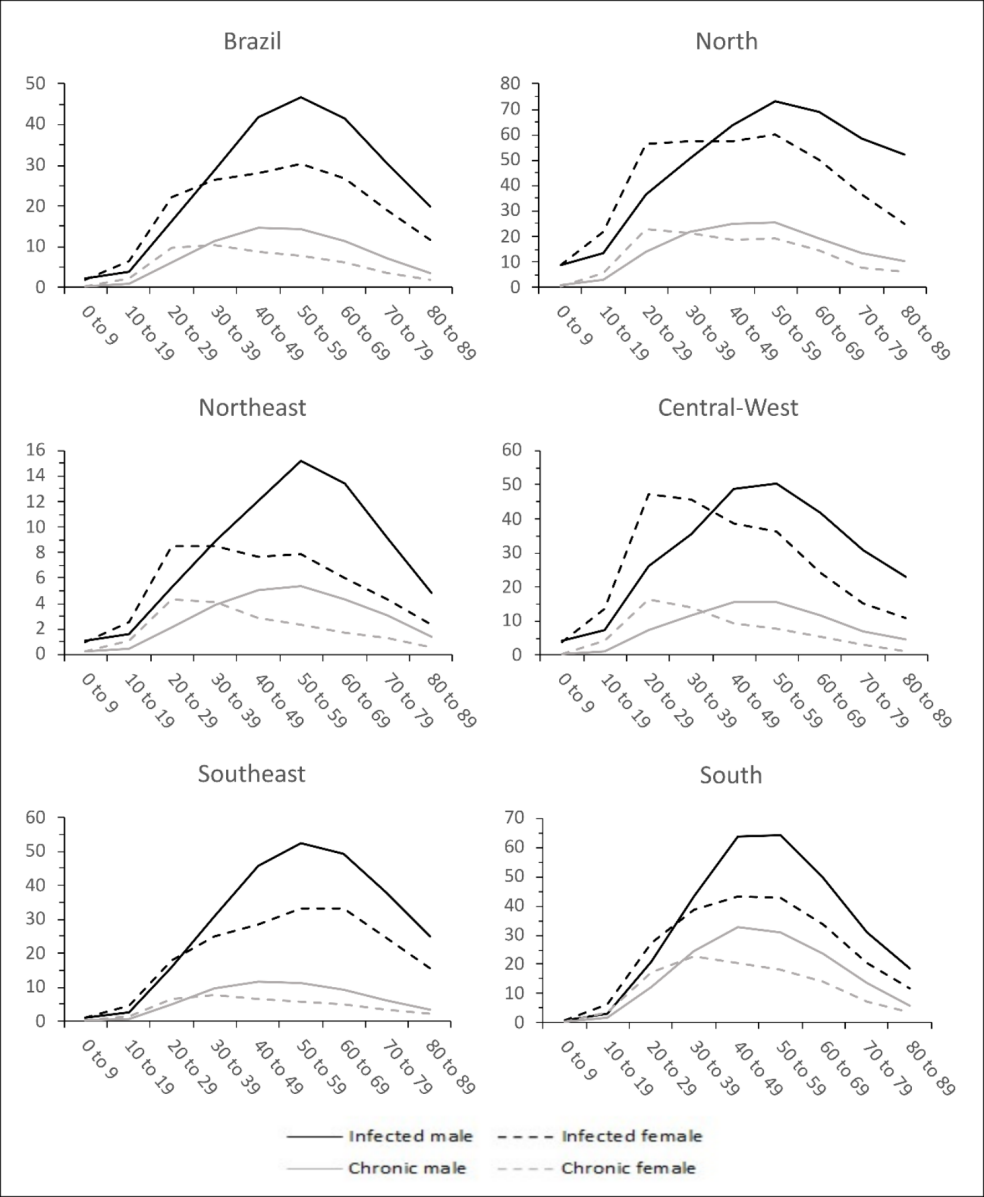
Detection rate of Hepatitis B cases per 100,000 inhabitants by age group from 2007 to 2018 for gender (male full line, female traced line), case definition (black = HBV *Infected* cases, grey = *Chronic* cases) and Brazilian regions

Figure 4 presents regional differences of the gender related HBV *Infected* and *Chronic* age-distributions from 2007 to 2018.

In Fig. 4, two patterns arise and relate to Brazilian regions. In the first pattern, seen in South and Southeast regions, the gender related age-distribution of HBV is very similar, rising up to 40–50 years old, plateauing until 60–70 years and decreasing for older ages. However, one difference worth describing is that men tend to keep the rise in infection rates for a longer age than women, whose infection rate diminishes from 20 to 25 years on.

The second pattern, seen in North, Northeast and Central-West, shows a considerable shift to the left on the age-related distribution of females as compared to males for ages below 30–40 years, being more pronounced bellow 30 years.

In addition, South and Southeast regions (first pattern described above), age difference between males and females are smaller as compared to North, Northeast or Central-West (second pattern above) for any HBV infection status. The most pronounced age difference occurs in *Chronic* infections in Northeast, 7.38 years (95% CI, 6.93–7.83), and Central-West 7.62 years (95% CI, 7.19–8.06), in contrast to *Chronic* infections in South, 3.76 years (95% CI 3.52–4.00) and Southeast, 3.71 years (95% CI 3.47–3.95). See table S3 of Supplementary Material for details.

Age differences on the mean age at notification by region and gender shows that people living in the North, Northeast and Central-West regions are infected earlier in life than South and Southeast. The most pronounced finding is the age difference of 10.3 years (95% CI, 10.04 to 10.56) for *Chronic* females and 7.88 years (95% CI, 7.63 to 8.13) for *Chronic* males when comparing North region with Southeast region. For the complete analysis see table S4 of Supplementary Material.

## Discussion

In Brazil, as in other countries, national regulations define which diseases are of compulsory notification and how to report them to the official notification system. Infection diseases surveillance systems are essential to guide health politics on national and regional scales but have limitations due to under notification. Several different reasons contribute to this, like failure in diagnosing the disease, in reporting the occurrence of disease to local health authorities, in limited technical or administrative structures, limiting the information flow between local and national systems, among others [20, 21].

Under notification may lead to under estimations of the true incidence or prevalence of a given disease. However, a careful analysis of the reported cases allows the identification of space, time and/or age related variations in the notification rate that can indicate changes in infection dynamics [21].

The Brazilian viral hepatitis notification system exists since 1998. During implantation, from 1998 to 2004, several improvements occurred allowing a better performance of the system as a whole. From 2007 to 2018, the system became stable and, consequently, changes in epidemiologic parameters of HBV infection may reflect changes in real infection dynamics.

Trend analysis estimates that Brazilian HBV incidence are stable from 2007 to 2018, but decreases among individuals younger than 39 years from 2013 onwards. In addition, incidence of *Acute* cases decreased for all age groups in the analyzed period (Fig. 1). Brazilian vaccination program and control measures improvement are probable explanations for these two findings.

In 1989, Brazilian National Vaccine Program initiated Hepatitis B routine vaccination for children (younger than ten years old) living in the endemic Amazon region [22]. In 1998, it implemented nationwide Hepatitis B newborn vaccination, with three doses at 0, 1 and 6 months of age, achieving nearly 98% of vaccine coverage in the following years [23, 24]. From 2003 on, the program expanded to reach people under 49 years old and in 2016 became universal, meaning that any individual have access to HBV vaccination offered by SUS. In addition, susceptible pregnant women are vaccinated on their first pre-natal consultation or at delivery together with the newborn.

In addition, other important hepatitis control measures adopted by Brazilian Health Authorities derive from the HIV/AIDS Brazilian control program, promoting safer sex practices and delivering condoms syringes and needles, under certain conditions, to at risk populations since early 2000 [24].

The effects of nationwide vaccination program and control measures in the incidence of HBV infection is also described in countries that, as Brazil, implemented Hepatitis B vaccination program around the year 2000 [24].

As mentioned in Results, the proportion of *Chronic*, *Acute* and *Resolved* cases differs greatly among regions. South and Northeast regions have the largest proportions of *Chronic* cases. This results deviates from the national proportion of *Chronic* cases and suggests that the burden of HBV *Chronic* infection is greater in this regions. The results of the Global Burden of Disease in Brazil [25] shows that Mortality Rates and Years of Life Lost (YLL) due to cirrhosis and liver cancer, both conditions related to Chronic Hepatitis B, are greater in males of the Northeast and Southeast regions.

The higher burden associated to HBV infection in these regions can be explained by HBV genotypes. To date, 10 HBV genotypes (A to J) have been identified, been genotype A and C associated with increased risk of chronicity and genotypes C and D with increased risk of liver cancer [26]. In Brazil, the most prevalent genotype is A (58.7%) followed by D (23.4%) and F (11.3%), however its distribution differs among Brazilian regions. In North and Northeast, genotype A is the most prevalent (71.6% and 65% respectively), while in South genotype D is the most prevalent (78.9%) [27]. This could explain the higher proportion of chronic HBV found in South and Northeast regions in SINAN database and the results of the Global Burden of Disease in Brazil [25].

As long as chronic Hepatitis B is considered, the notification increase for those above 40 years-old seen from 2007 to 2018 in Brazil has also been described in China and USA [28, 29]. As discussed in those articles, the improvement of healthcare can explain these findings as it facilitates diagnosis and decreases mortality rates associated with chronic hepatitis B complications, such as liver cancer and cirrhosis [30].

Similarly to those countries, Brazil has also experienced a reduction in mortality rates of cirrhosis and liver cancer in the last decade [30, 31] consequently increasing life expectancy of the chronically infected. This is a direct consequence of the investment increase in Hepatitis B care [23], granted by Brazilian government, comprising access to testing, consultation with hepatitis specialists, antivirals delivery, and laboratory and image follow-ups.

However, the hepatitis B program are limited and, therefore, can diagnose and treat yearly only a fraction of people chronically infected with hepatitis B. Moreover, untreated chronically infected people may infect new partners who can evolve to unnoticed chronic infections. In addition, there is a suggestion that HBV vaccination immunity can wane after long periods of time [32], what could contribute to a further increase in chronic infection pool. As a result, in spite of reducing this pool every year by the improvement on control program activities, it shall remain with a substantial number of people for the next decades.

As described, individuals from South and Southeast acquire Hepatitis B later in life when compared to the other regions. Also, males and females age-related infection rates in this regions are similar, in contrast to North, Northeast and Central-West regions. South and Southeast regions have lower Gini index, lower infant mortality rate, lower women fecundity rate, higher maternal age at firstborn and higher life expectancy at birth when compared to North, Northeast and Central-West regions [11, 33]. This better socio-demographic profile of South and Southeast could explain the patterns described in Fig. 4.

This study have strengths and limitations. As off strengths, the proposed case definitions are less restrictive allowing the analysis of a much larger number of cases, although keeping a strict correlation with official HBV status classification (Table 2). The analysis performed to estimate temporal trends of Hepatitis B reported cases follows the methodology adopted by the NIH National Cancer Institute to estimate cancer trends, which was also applied in infectious diseases, specifically in Hepatitis B, by other authors [28, 29]. This similarity allows a proper comparison among different world regions.

In addition, the analysis performed in this manuscript highlights the use of gender related age distribution, a parameter rarely explored in literature, but of upmost importance for understanding the infection dynamics in a given population.

Limitations of this work relate to the quality of available data. The Brazilian Viral Hepatitis database includes only a fraction of the total HBV *Infected* individuals, suffering from under-notification. In addition, many observations are incomplete, while some are duplicated, making troublesome, but extremely necessary, a careful revision of data extracted from SINAN. In addition, even with this careful approach the completeness and correction of data cannot be fully warranted. Notwithstanding, as shown in this work, it is possible to recover significant information on disease dynamics even from such incomplete notification database registries.

## Conclusion

The analysis performed in this article demonstrates changes in notification rates of Hepatitis B, possibly reflecting differences in national health politics, vaccination programs and universal access to treatment. In addition, regional differences suggest that North, Northeast and Central-West populations are at higher risk to acquire HBV infection earlier in life and develop chronic infection; therefore, vaccination programs should prioritize these regions. Finally, gender differences points to a higher female vulnerability that have to be taken into account on control programs in the less developed Brazilian regions.

## Electronic supplementary material

Below is the link to the electronic supplementary material.

Supplementary Material 1

## Data Availability

The datasets generated and/or analysed during the current study are available in the SINAN - Sistema Nacional de Agravos de Notificação repository, http://portalsinan.saude.gov.br/ - accessed in 06-11-2020.

## Abbreviations

HBV: Hepatitis B Virus.
MoH: Brazilian Ministry of Health.
SUS: Sistema Único de Saúde.
DCCI: Department of Chronic Illness and Sexually Transmitted Infections.
SINAN: Brazilian National Notifiable Disease Reporting System.
BIC: Bayesian Information Criteria.
APC: Annual Percent Change.
AAPC: Average Annual Percent Change.
