# Supplementary material for: Regional differences and temporal trend analysis of Hepatitis B in Brazil
Source: BMC Public Health. 2022 Oct 17;22:1931. doi: 10.1186/s12889-022-14296-1 (PMC9578265; doi:10.1186/s12889-022-14296-1)
Supplement: Supplementary file 1 — Supplementary Material 1 [file 12889_2022_14296_MOESM1_ESM.docx]

| **Infection Status** | **AAPC (2007-2018)**  **with 95% CI** | **APC (periods)**  **with 95% CI** |
| --- | --- | --- |
| Infected | -1.6 (-6.0 to 2.9) | **2007 to 2012: 9.1 (3.6 to 14.8)** |
|  |  | 2012 to 2016: -4.6 (-15.0 to 7.0) |
|  |  | 2016 to 2018: -19.2 (-35.8 to 1.8) |
| Acute | **-5.3 (-9.9 to -0.4)** | **2007 to 2018: -5.3 (-9.9 to -0.4)** |
| Chronic | 1.7 (-0.4 to 3.8) | **2007 to 2012: 8.1 (3.8 to 12.5)** |
|  |  | **2012 to 2018: -3.3 (-6.2 to -0.3)** |
| Resolved | -3.7 (-8.0 to 0.8) | **2007 to 2012: 10.6 (4.9 to 16.6)** |
|  |  | 2012 to 2016: -5.5 (-16.0 to 6.3) |
|  |  | **2016 to 2018: -29.2 (-44.1 to -10.4)** |

**Table S1:** Results of Brazilian Hepatitis B reported cases temporal trend analysis. In bold values that achieved statistical significance (P<0.05).

| **Region** | **HBV Status** | **Absolute cases** | **Percentage (%)** | **95% Confidence Interval (%)** |
| --- | --- | --- | --- | --- |
| Brazil | Infected | 475,759 | 100 | - |
|  | Acute | 13,669 | 2.87 | 2.83 - 2.92 |
|  | Chronic | 161,283 | 33.90 | 33.7 - 34.03 |
|  | Resolved | 300,807 | 63.23 | 63.09 - 63.36 |
| North | Infected | 73,474 | 100 | - |
|  | Acute | 1,122 | 1.53 | 1.44 - 1.62 |
|  | Chronic | 25,670 | 34.94 | 34.59 - 35.28 |
|  | Resolved | 46,682 | 63.54 | 63.19 - 63.88 |
| Northeast | Infected | 38,427 | 100 | - |
|  | Acute | 1,983 | 5.16 | 4.94 - 5.38 |
|  | Chronic | 15,871 | 41.30 | 40.81 41.79 |
|  | Resolved | 20,573 | 53.54 | 53.04 - 54.04 |
| Central-West | Infected | 49,323 | 100 | - |
|  | Acute | 1,217 | 2.47 | 2.33 - 2.6 |
|  | Chronic | 14,537 | 29.47 | 29.07 - 29.88 |
|  | Resolved | 33,569 | 68.06 | 67.65 - 68.47 |
| Southeast | Infected | 218,320 | 100 | - |
|  | Acute | 5,862 | 2.69 | 2.57 - 2.8 |
|  | Chronic | 54,462 | 24.95 | 24.63 - 25.26 |
|  | Resolved | 157,996 | 72.37 | 72.05 - 72.69 |
| South | Infected | 96,215 | 100 | - |
|  | Acute | 3,485 | 3.62 | 3.50 - 3.74 |
|  | Chronic | 50,743 | 52.74 | 52.42 - 53.05 |
|  | Resolved | 41,987 | 43.64 | 43.33 - 43.95 |

**Table S2:** Absolute number of HBV cases by infection status and regional proportion with 95% confidence interval.

|  | Infected | | | Acute | | | Chronic | | | Resolved | | |
| --- | --- | --- | --- | --- | --- | --- | --- | --- | --- | --- | --- | --- |
|  | Male | Female | ***ΔAge (95% CI)*** | Male | Female | ***ΔAge (95% CI)*** | Male | Female | ***ΔAge (95% CI)*** | Male | Female | ***ΔAge (95% CI)*** |
| BR | 44.48 | 41.32 | ***3.16 (3.07 - 3.24)*** | 41.60 | 39.90 | ***1.69 (1.21 - 2.18)*** | 43.24 | 38.42 | ***4.82 (4.68- 4.96)*** | 45.82 | 42.95 | ***2.39 (2.27 - 2.50)*** |
| N | 38.81 | 34.90 | ***3.9 (3.66 - 4.14)*** | 39.95 | 36.00 | ***3.94 (2.2 - 5.59)*** | 39.62 | 35.11 | ***4.5 (4.1 - 4.84)*** | 38.32 | 34.77 | ***3.54 (3.22 - 3.87)*** |
| NE | 42.60 | 37.12 | ***5.48 (5.16 - 5.79)*** | 39.57 | 37.64 | ***1.93 (0.56 - 3.29)*** | 42.46 | 35.07 | ***7.38 (6.93 - 7.83)*** | 43.17 | 38.76 | ***4.41 (3.94 - 4.88)*** |
| CO | 41.17 | 36.18 | ***4.99 (4.73 - 5.25)*** | 40.23 | 35.68 | ***4.54 (3.02 - 6.07)*** | 42.20 | 34.57 | ***7.62 (7.19 - 8.06)*** | 40.72 | 36.93 | ***3.79 (3.45 - 4.12)*** |
| SE | 46.69 | 45.20 | ***1.48 (1.36 - 1.61)*** | 41.88 | 41.40 | ***0.48 (-0.26 - 1.24)*** | 44.02 | 40.30 | ***3.71 (3.47 - 3.95)*** | 47.87 | 46.99 | ***0.88 (0.73 - 1.03)*** |
| S | 45.46 | 43.19 | ***2.28 (2.1 - 2.46)*** | 43.17 | 42.18 | ***0.98 (0.05 - 1.91)*** | 44.54 | 40.77 | ***3.76 (3.52 - 4.00)*** | 46.79 | 46.03 | ***0.76 (0.48 - 1.04)*** |

**Table S3:** Gender differences on the mean age at first notification for each HBV status by region, according to **HBV Class 2**.

*ΔAge = Mean Age for males – Mean Age for females* (difference 95% CI interval). All differences were statistically significant, with a single exception (underlined).

| **HBV infection status** | **Region 1** | **Region 2** | **Diference (95% CI)** | |
| --- | --- | --- | --- | --- |
|  |  |  | **Males** | **Females** |
| **Hepatitis B** | **North** | **Northeast** | -3.79 (-4.15 to -3.42) | -2.22 (-2.61 to -1.83) |
|  |  | **Southeast** | -7.88 (-8.13 to -7.63) | -10.3 (-10.56 to -10.04) |
|  |  | **South** | -6.66 (-6.94 to -6.37) | -8.28 (-8.59 to -7.98) |
|  |  | **Central-West** | -2.36 (-2.71 to -2.01) | -1.27 (-1.62 to -0.92) |
|  | **Northeast** | **Southeast** | -4.09 (-4.4 to -3.77) | -8.08 (-8.43 to -7.73) |
|  |  | **South** | -2.87 (-3.21 to -2.52) | -6.07 (-6.44 to -5.69) |
|  |  | **Central-West** | 1.43 (1.04 to 1.83) | 0.95 (0.53 to 1.36) |
|  | **Southeast** | **South** | 1.22 (1 to 1.44) | 2.02 (1.77 to 2.26) |
|  |  | **Central-West** | 5.52 (5.22 to 5.82) | 9.03 (8.72 to 9.33) |
|  | **South** | **Central-West** | 4.3 (3.97 to 4.63) | 7.01 (6.67 to 7.35) |
| **Acute** | **North** | **Northeast** | *0.38 (-1.49 to 2.25)* | *-1.63 (-3.97 to 0.7)* |
|  |  | **Southeast** | -1.93 (-3.56 to -0.3) | -5.39 (-7.44 to -3.34) |
|  |  | **South** | -3.22 (-4.94 to -1.49) | -6.18 (-8.31 to -4.05) |
|  |  | **Central-West** | *-0.28 (-2.42 to 1.87)* | *0.32 (-2.15 to 2.79)* |
|  | **Northeast** | **Southeast** | -2.31 (-3.58 to -1.04) | -3.76 (-5.46 to -2.06) |
|  |  | **South** | -3.6 (-4.98 to -2.21) | -4.55 (-6.34 to -2.75) |
|  |  | **Central-West** | *-0.66 (-2.54 to 1.23)* | *1.95 (-0.23 to 4.14)* |
|  | **Southeast** | **South** | *-1.29 (-2.33 to 0.25)* | *-0.79 (-2.19 to 0.62)* |
|  |  | **Central-West** | 1.65 (0.01 to 3.3) | 5.71 (3.84 to 7.59) |
|  | **South** | **Central-West** | 2.94 (1.2 to 4.68) | 6.5 (4.53 to 8.46) |
| **Chronics** | **North** | **Northeast** | -2.84 (-3.39 to -2.28) | *0.04 (-0.53 to 0.61)* |
|  |  | **Southeast** | -4.4 (-4.81 to -3.99) | -5.19 (-5.63 to -4.75) |
|  |  | **South** | -4.92 (-5.33 to -4.5) | -5.66 (-6.1 to -5.22) |
|  |  | **Central-West** | -2.58 (-3.15 to -2) | *0.54 (-0.04 to 1.12)* |
|  | **Northeast** | **Southeast** | -1.56 (-2.05 to -1.08) | -5.23 (-5.75 to -4.71) |
|  |  | **South** | -2.08 (-2.57 to -1.59) | -5.7 (-6.22 to -5.18) |
|  |  | **Central-West** | *0.26 (-0.37 to 0.9)* | *0.5 (-0.14 to 1.15)* |
|  | **Southeast** | **South** | -0.52 (-0.84 to -0.2) | -0.47 (-0.84 to -0.1) |
|  |  | **Central-West** | 1.82 (1.31 to 2.34) | 5.73 (5.2 to 6.26) |
|  | **South** | **Central-West** | 2.34 (1.82 to 2.86) | 6.2 (5.67 to 6.73) |
| **Resolved** | **North** | **Northeast** | -4.85 (-5.36 to -4.35) | -3.99 (-4.54 to -3.45) |
|  |  | **Southeast** | -9.55 (-9.88 to -9.23) | -12.22 (-12.55 to -11.89) |
|  |  | **South** | -8.47 (-8.89 to -8.06) | -11.26 (-11.69 to -10.82) |
|  |  | **Central-West** | -2.4 (-2.85 to -1.94) | -2.16 (-2.6 to -1.72) |
|  | **Northeast** | **Southeast** | -4.7 (-5.14 to -4.26) | -8.23 (-8.72 to -7.74) |
|  |  | **South** | -3.62 (-4.13 to -3.11) | -7.26 (-7.83 to -6.7) |
|  |  | **Central-West** | 2.45 (1.91 to 3) | 1.83 (1.26 to 2.41) |
|  | **Southeast** | **South** | 1.08 (0.75 to 1.41) | 0.97 (0.6 to 1.33) |
|  |  | **Central-West** | 7.16 (6.78 to 7.53) | 10.06 (9.68 to 10.44) |
|  | **South** | **Central-West** | 6.07 (5.62 to 6.53) | 9.09 (8.63 to 9.56) |

**Table S4:** Regional differences in the mean age of first notification, by gender and case definition (according to Class2). Differences refers to ***Region 1 - Region 2***. In *italic* non-significant differences (*p* ≥ 0.05).
